# Electrochemical valorization of waste activated sludge for short-chain fatty acids production

**DOI:** 10.3389/fchem.2022.974223

**Published:** 2022-08-30

**Authors:** Maasoomeh Jafari, Gerardine G. Botte

**Affiliations:** Chemical and Electrochemical Technology and Innovation Laboratory, Department of Chemical Engineering, Edward E. Whitacre Jr. College of Engineering, Texas Tech University, Lubbock, TX, United States

**Keywords:** volatile fatty acids, transition metals, sludge electrolysis, ammonia synthesis, sludge revalorization, electrochemical digestors

## Abstract

A tremendous amount of waste activated sludge (WAS) ends up in landfilling even after a substantial retention time during anaerobic digestion. This leftover activated sludge is an organic-rich material with the high potential to produce value-added chemicals such as short chain fatty acids (SCFAs). In the present study, a novel electrochemical conversion of activated sludge (E-WAS) was carried out on the surface of non-precious electrodes (nickel, stainless-steel and copper) in alkaline media at low applied potential and temperature. Cyclic voltammetry showed that Cu (II)/Cu (III) and Ni (II)/Ni(III) redox couple catalyzed the WAS oxidation reaction to produce SCFAs and hydrogen. The results revealed that Cu(II)/Cu(III) has higher catalytic oxidation capability towards SCFAs. Yields of 48.7, 21.4, and 14.6 mg SCFAs per g of volatile solids were achieved by using copper, nickel and stainless-steel as working electrodes, respectively. Post analysis characterization techniques indicate that copper oxide films lead to WAS oxidation. Total volatile solid removal of 30% was obtained at 35°C and 1.65 V in 0.2 M NaOH after 2 h of operation in an electrochemical digestor with copper electrodes which is more efficient than a conventional alkaline treatment (24 h, 55%, 1M NaOH). Ammonia was produced as the by-product of E-WAS oxidation. The highest amount of ammonia (250 mg L^−1^) was obtained by using nickel as the working electrode after 2 h operation at 35°C and 1.35 V applied potential. The change in WAS morphology revealed that the copper oxide film is an effective electrocatalyst for WAS disinfection.

## Graphical Abstract

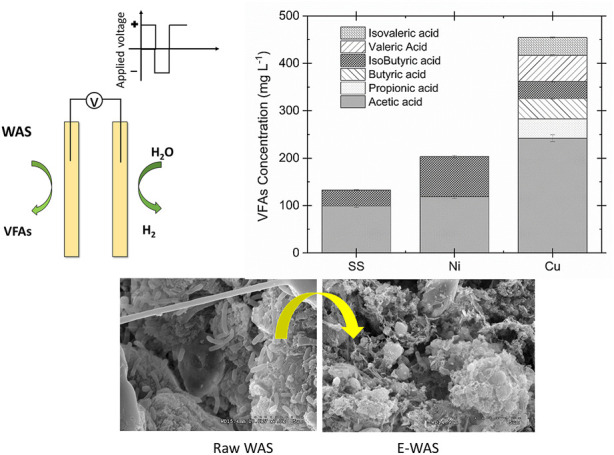

## Introduction

Waste activated sludge (WAS) is the major byproduct of municipal wastewater treatment plants (WWTPs). Management and disposal of WAS create challenges for WWTPs such as high energy consumption and operational costs. Approximately, over 12 million tons per year of sludge are produced in the US (Seiple et al., 2017) consuming more than 30 TWh per year which is equal to $2 billion annual electricity cost for treatment of WAS implementing conventional treatment process (Lemar and De Fontaine, 2017). About 50–60% of the operational costs in wastewater treatment plants are dedicated to sludge treatment and disposal. More importantly, WAS contains large amounts of pathogens that can cause serious environmental hazards if inappropriately disposed and treated (Zhao et al., 2018). Hence, it is critical to find sustainable alternative ways to treat WAS.

WAS contains organic material like lignocellulosic waste that could be converted to produce high value chemicals such as volatile fatty acids. Volatile fatty acids (VFAs) are short chain carboxylic acids that contain two to six carbon atoms. VFAs are important precursors to produce a wide range of value-added chemicals such as biopolymers (i.e., polyhydroxyalkanoates–PHAs) and other valuable products including biofuel. The price range of VFAs is between $600–3,815 t^−1^ depending on the number of carbon atoms in the VFAs. The global market demand for VFAs in 2020 was 18,500 kt and it is expected that it will increase at an annual rate of 3% per year for the next 3 years (Ramos-Suarez et al., 2021). These valuable compounds are currently produced by petrochemical routes (such as methanol carbonylation, oxidation of propane, and oxidation of butyraldehyde) which are energy, cost, and carbon intensive processes (Ramos-Suarez et al., 2021); for instance, 3.3 t of CO_2_ are generated for every ton of acetic acid produced (Veluswamy et al., 2021). The waste and wastewater treatment industries have a unique opportunity to meet the increasing market demand for VFAs with a lower carbon footprint (for example, up to 85 mg of VFAs are produced per g of volatile solid (VS.) after 6 days of fermentation in anaerobic digestion (AD) of WAS) while enabling new pathways to convert waste into value products towards a circular economy (Ji et al., 2010; Bruni et al., 2021).

Anaerobic digestion is the conventional method for WAS treatment and energy recovery in the form of biogas (methane and carbon dioxide), however, methane is a low value product ($150 per ton) (Xu et al., 2014; Liu et al., 2018; Chiappero et al., 2019; Li et al., 2019; Siami et al., 2020). The sluggish WAS hydrolysis is the rate limiting step in the AD and results in a low VFAs production rate (100 mg L^−1^ per day). Furthermore, the long residence time of the WAS in the AD ends up converting the VFAs produced during fermentation to biogas. A recent study showed that the net profit of VFAs production in AD is approximately three times higher than biogas with shorter residence times (7 days for VFAs, 25 days for biogas) (H. Liu et al., 2018). It was reported that an alkaline pH has a predominantly positive effect on VFA production. Studies have also shown that higher VFA production rates of 300 mg L^−1^ per day than the production rate in anaerobic digestion (100 mg L^−1^ per day) are possible from WAS under alkaline conditions (Ma et al., 2016).

Electrochemical treatment is a robust, scalable, and environmentally friendly approach that has been widely studied for wastewater treatment processes. For instance, to improve sludge dewaterability (She et al., 2016; Masihi and Badalians Gholikandi, 2018; Cai et al., 2019; Lu et al., 2022), to enhance biogas recovery from anaerobic digestion (Barrios et al., 2021; Hao Huang et al., 2021), for sludge conversion and pathogen removal (Zeng et al., 2019; Jafari and Botte, 2021; Martínez-Huitle and Brillas, 2021), for pollutant removal (Qiao and Xiong, 2021), and for nutrient recovery such as nitrogen and phosphorous (Arslan et al., 2017; Yang et al., 2020; Xu et al., 2021). Our previous research has shown that electrochemical treatment of WAS under alkaline conditions could improve sludge defragmentation and solubilization (Jafari and Botte, 2021), electrochemically enhanced digestors. To our knowledge, no prior studies have examined the electrooxidation of the waste activated sludge for VFAs production at low operating voltages and short residence times. Other studies which implemented electrochemical routes for WAS treatment operated at high voltage e.g., 11–20 V and long operating times (Charles et al., 2013; Yu et al., 2014; Masihi and Badalians Gholikandi, 2018) a recent summary of the approaches can be found in the literature (Zeng et al., 2022). In this paper, we demonstrate a novel process to produce short fatty acids from waste activated sludge via electrolysis in mild alkaline media implementing copper, nickel, and stainless-steel electrodes. Copper and nickel were selected for the study as they have been used in the electrocatalysis of other organic compounds, such as such as urea, lignin, glucose, and chitin (Jafarian et al., 2009; Eshghi and Kheirmand, 2018; Zhao et al., 2021). On the other hand, the combination of high corrosion resistance and excellent mechanical strength, makes stainless steel an ideal candidate for electrochemical processes if catalytically active (Madih et al., 2020; Todoroki and Wadayama, 2021). The electrolysis of waste activated sludge (E-WAS) enables biosolid solubilization and production of chemicals of value at low cell voltage and temperature under mild alkaline conditions. E-WAS would enable process intensification in municipal wastewater treatment plans and could transform sludge management operation from cost model to revenues generation leading towards circular economies.

## Experimental

### Materials

Stainless steel mesh 316 (316 woven, 10 × 10 mesh size, 58% opening, cat no, 9656T13, McMaster-Carr), Nickel mesh (15Ni (201)17, thickness 0.762 mm, Dexmet) and copper foil (110copper, cat no. 8963K205, thickness 0.0210 inch, McMaster-Carr) were used as materials for electrodes fabrication. Sodium hydroxide (NaOH 98%, Fisher Scientific) was used as electrolyte in the electrochemical cell. Phosphate buffer solution (0.1 M, Sigma Aldrich), glutaraldehyde ampule (8%, Electron Microscopy Sciences), and reagent grade water (REF, 9,800–3, NERL) were used for sample preparation of biomass for scanning electron microscopy analysis. Sulfuric acid (95–98%, Certified ACS plus, Fisher Scientific) was used for liquid sample acidification to quantify the VFAs by Gas chromatography- Flame ionization detector (GC-FID, Agilent 8,890).

### Waste activated sludge sample

Samples were obtained from the municipal wastewater treatment plant located in Lubbock, Texas. The sludge was collected after the centrifuge step before preparation for disposal to landfill. The dewatered sludge (DS) with approximately 80% moisture was transferred to the lab and stored at 4°C before characterization and electrochemical treatment.

### Analytical techniques

The liquid samples after treatment were centrifuged (Thermo Scientific, Multifuge X1R) at 4,500 rpm for 15 min. The 5 ml of supernatant liquid was transferred to a 15 ml centrifuged vial (Fisher brand) and acidified with 1 M sulfuric acid to obtain pH 2. The acidified sample was centrifuged at 4,500 rpm for 1 min to remove the precipitant solid after the acidification step. The acidified liquid was filtered with a 0.45 μm cellulose acetate membrane syringe filter (G.E. Healthcare Whatman^®^ Puradisc 30 syringe filters, catalog number: 10462100) before injection to GC-FID (Agilent 8,890) for VFAs analysis. Helium (Ultra high purity, Praxair) was used as carrier gas and was controlled in constant flow mode at a linear velocity of 42 cm s^−1^. The inlet temperature was set at 280°C with a spilt ratio of 50:1. The oven temperature was programmed to start at 120°C (held for 2 min), followed by ramping at 5°C min^−1^–140°C (held for 3 min); and finally ramping at 20°C min-1–250°C (held for 10 min). Sample injection volumes were kept constant at 0.1 µL. The FID temperature was set at 280°C. A DBFATWAX Ultra (Agilent, part number, G3903-63008, 30 m, 0.25 mm, 0.25 µm) column was utilized for VFAs quantification. VFAs standard mixtures (Sigma Aldrich, CRM46975) were used for the GC-FID calibration. Gases generated after E-WAS were analyzed by gas chromatography (Shimadzu-GC 2014) with a thermal conductivity detector (TCD). The gas chromatogram of E-WAS samples is shown in the supporting information as Supplementary Figure S5. A volume of 1 ml of gas sample was injected to the GC by using a gas tight syringe. The oven temperature was held at 60°C for 5 minutes followed by a ramp of 10°C min^−1^ up to 200°C and then held for 5 minutes. The injector valve and detector temperatures were 125 and 200°C, respectively. Helium (Ultra high purity, Praxair) was used as the carrier gas with a flow rate of 20 ml min^−1^.

Thermal analysis of the samples was conducted using thermogravimetric analysis (TGA, STAPT 1600, Linseis) to determine volatile solid removal efficiency after electrochemical oxidation of WAS. First, a blank test was performed (empty alumina crucible Linseis, 70 μL) to investigate the empty pan thermal behaviour during the operating condition. The empty alumina crucible was inserted into the TGA furnace, and the temperature was ramped up from 25 to 600°C with a ramp rate of 20°C min^−1^ under nitrogen (99.99%, Airgas) atmosphere followed by cooling down to 25°C. For volatile solid determination approximately 20 mg of the WAS sample before or after electrochemical oxidation was put into 70 μL alumina crucible with the temperature profile like a blank test. The nitrogen flow rate was set to 50 ml min^−1^ during the thermal analysis test. The morphological structure of the WAS samples was characterized using Field-emission Scanning Electron Microscope (FE-SEM, Hitachi S-4300). The raw solid WAS sample and solid after centrifuging were prepared using a critical point drying instrument (autosamdri-814, Tousimis research corporation, details of sample preparation presented in Supplementary Information, Supplementary Table S1) and spotter coated with platinum. The copper oxide layer formed on the surface of the copper substrate (see *Analytical techniques*) was characterized by X-Ray diffraction (XRD, Rigaku Smart Lab 3 kW XE X-ray diffractometer), Raman spectroscopy (T-Raman microscope module/SENTERRA dispersive Raman microscope spectrometer (Bruker Optics)) and FE-SEM (Hitachi-4700) microscopy. XRD pattern was obtained for the 2θ range of 20–80° and the Raman measurement was performed at an excitation wavelength of 532 nm, resolution of 3–5 cm^−1^, and 1,200–50 spectra absorption range. The concentration of ammonia in the samples was measured with an ammonia ion-selective electrode (Thermo Scientific, Orion 9512BNWP).

### Fabrication and preparation of electrodes

The stainless-steel (SS), nickel (Ni), and copper (Cu) electrodes were cut (using metal scissors) to dimensions 5 cm × 5 cm, for geometric surface area of 25 cm^2^. The electrodes were sonicated in an Ultrasonic bath (Branson 2,800) first with isopropanol solution (ACS grade, Fisher Scientific) and then with deionized water (DI) water for 10 min each at high frequency to obtain a clean surface. Stainless steel and nickel wires were used as the current collector for the stainless steel and nickel mesh, respectively. The current collectors were spot welded on the stainless steel and nickel mesh for 1 s using a spot welder (Stark Professional Portable Spot Welder Machine). The copper electrode was cut with an extended part to serve as current collector. To study the copper electrode surface for electrochemical conversion of WAS, the electrode was cut in a way to have the current collector compatible with the electrode. To avoid galvanic corrosion, the current electrodes were made of the same material as the electrodes, for example, it has been reported that welding titanium wires to copper electrodes leads to galvanic corrosion (Du et al., 2014). Detailed information about the dimensions of the electrodes is described in the supplementary section (Supplementary Figure S1).

### Electrochemical cell, experimental setup, and process

To identify the oxidation state of the electrode surface, cyclic voltammetry tests (CV) were carried out for all the electrodes at 25°C in 250 ml beakers (Fisher brand). Identical electrodes (Ni, SS, and Cu) were used for the working and counter electrodes and Hg/HgO (Koslow Scientific) was used as the reference electrode. Both working and counter electrodes were polished with sandpaper (400 and 600 grits) followed by sonication in DI water for 10 min at high frequency in Ultrasonic bath (Branson 2,800). The reference electrode was supported using a Luggin capillary filled with the supporting electrolyte (0.2 M NaOH). A volume of 200 ml of 0.2 M NaOH solution was used for each CV experiment. The cyclic voltammograms experiments (CVs) were obtained between −1.2 and 0.8 V vs. Hg/HgO at a scan rate of 50 mVs^−1^. In all the cases the sustained periodic state was achieved after 10 sweeps.

Electrolysis experiments were performed in a single-chamber electrolysis cell fabricated with acrylic plexiglass (with an inlet and outlet diameter of 0.635 cm) as described by Jafari and Botte (Jafari and Botte, 2021). Slurry samples/mixtures consist of biosolid 83 g L^−1^ with 0.2 M NaOH. The slurry mixture was fed to the electrolysis cell with a flow rate of 401 ml min^−1^ using a peristaltic pump (Cole Parmer Instruments, Catalog No. 7553–22) connected to a speed controller (Masterflex). The biosolid slurry was kept in the feed reservoir at 35–55°C and well mixed at 600 rpm using a magnetic stir bar (PTFE, 2.5 cm) for 5 min before feeding to the electrolysis cell to obtain a homogenous feed slurry. A total volume of 600 ml slurry was used in each experiment. The homogeneous slurry was fed to the electrochemical cell for electrochemical oxidation. The schematic of the electrolysis system is shown in Supplementary Figure S2.

The electrolysis cell was connected to a Gamry Interface 5000P for power supply. Electrolysis experiments were performed at control cell voltage using a polarity switch to reverse the cathode and anode with pulse times of 10 s. Polarity switching was implemented to reduce transport limitations and to mitigate blockage of active surface area for the electrochemical reaction, which is typical during the electrolysis of ammonia and nitrogenated compounds, as reported by Vitse et al. (2005). The pulse rate for polarity switching was kept constant; future experiments could involve the optimization of the pulsing time. The effect of operating temperature, cell potential and electrode type on volatile solids removal and short chain fatty acid production were investigated. The temperature of the electrochemical cell was controlled with a temperature controller (Econo Temperature controller 12125–14) within the range of 35–55°C. The ammonia and short chain fatty acid concentrations were analyzed after each experiment to determine the effectiveness of E-WAS for biomass conversion.

### Volatile solids removal efficiency and VFAs yield

Thermogravimetric analysis was conducted for the raw and the WAS after electrochemical treatment for the temperature range of 25–600°C to calculate the quantity of volatile solids (VS.). The total VFAs concentration was determined by GC-FID (Agilent, 8,990). The total VFAs yield, and total volatile solids removal efficiency were calculated by Eqs 1, 2.

$$\text{VS removal efficiency}=\frac{{\text{VS}}_{2}-{\text{VS}}_{1}}{{\text{VS}}_{1}}$$ (1)

$$\text{VFAs yield }\left({\text{mg gVS}}^{-1}\right)=\frac{\text{VFAs conc}.\;\times \text{V}}{{\text{VS}}_{1}}$$ (2)

Where the VS_2_, VS_1_, VFAs concentration, and V are the weight of total volatile solids after and before electrochemical oxidation, volatile fatty acid concentration (mg L^−1^), and total sample volume (L), respectively. The energy consumption of the E-WAS was calculated using Eqs 3, 4 per kg of TS (dried) and kg of VFA produced, respectively:

$$\text{Energy consumption }\left({\text{kWh}\text{kg}\text{TS}}^{-1}\right)=\frac{\text{I}\times V_{applied}\times t}{1000\times \text{TS}}$$ (3)

$$\text{Energy consumption }\left({\text{kWh}\text{kg}\text{TS}}^{-1}\right)=\frac{\text{I}\times V_{applied}\times t}{1000\times \text{VFAmass}}$$ (4)

Where the I, Vapplied, t, TS, and VFAmass are the current (A), applied potential (V), electrolysis time (h), total solids (kg) and mass of VFA produced (kg).

## Results and discussion

### Characterization of materials

**FIGURE 1:**
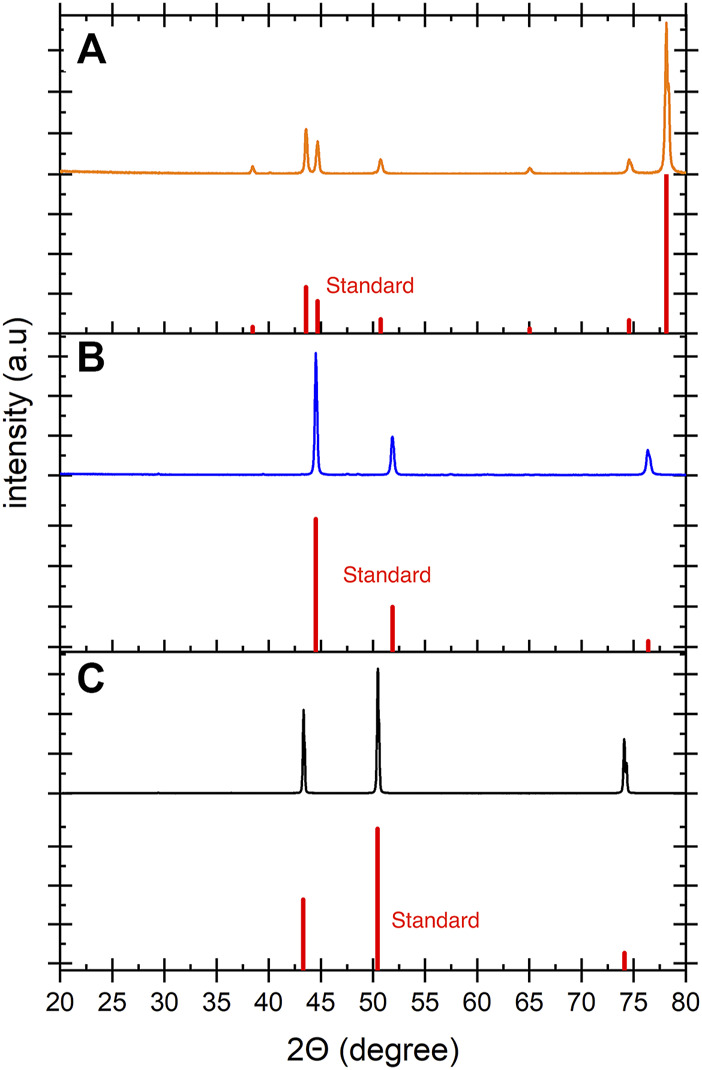
The XRD patterns of the **(A)** SS mesh, **(B)** Ni mesh and **(C)** Cu foil before E-WAS. The corresponding peaks confirm the crystal structure of the electrodes before electrolysis. The standard XRD patterns are shown in red.

The crystalline structures of the electrode materials were analyzed by XRD. Figure 1 shows XRD patterns of the electrodes used in this study. Stainless steel mesh (Figure 1A) showed peaks at 43.6°, 50.9°, and 74.7° which are correlated with the (111), (200) and (220) planes of austenite. The peaks at 38.5° and 78.1° correspond to the alumina sample holder (Dongsong RONG and Dongsong RONG, 2015). The nickel mesh (Figure 1B) showed peaks at 44.5°, 51.9° and 76.4° which correspond to the (111), (200) and (220) planes of the nickel (Ul-Hamid et al., 2015). Copper foil (Figure 1C) presents three peaks at 43.3°, 50.4° and 74.7° which are attributed to the (111), (200) and (220) planes of copper (Mardiansyah et al., 2018).

### Effect of electrode type on the WAS conversion

**FIGURE 2:**
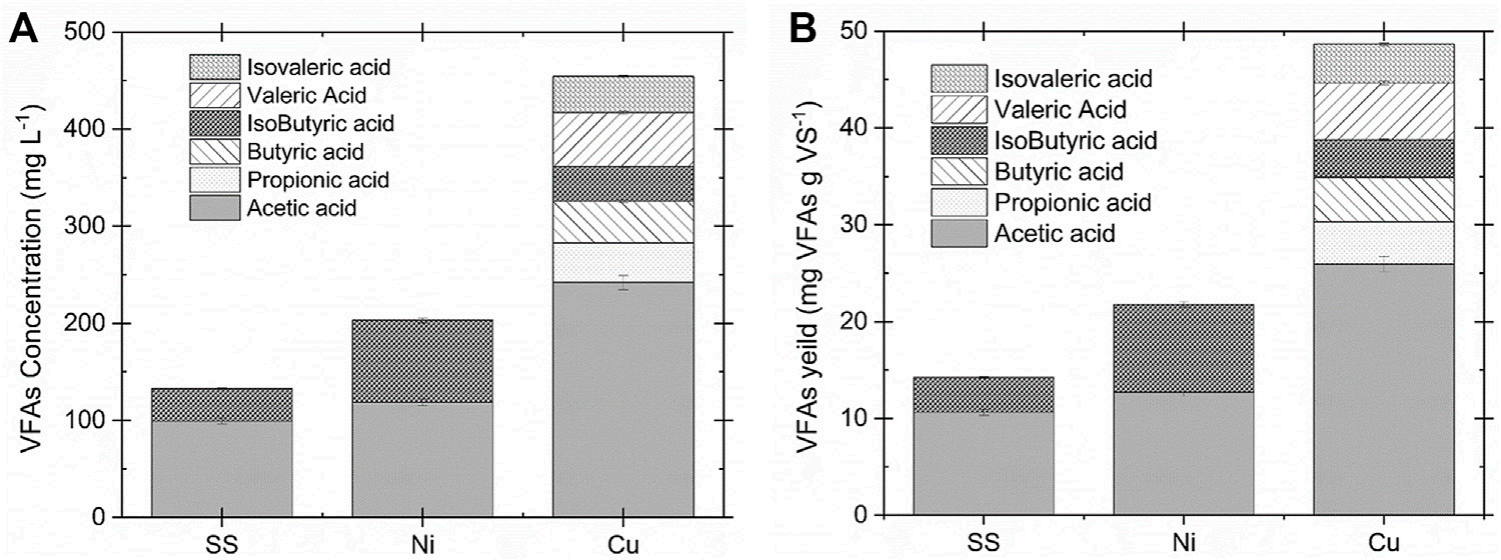
**(A)**VFAs concentration and **(B)** yield comparison for the SS, Ni, and Cu electrodes at 35°C and 1.35 V in 0.2 M NaOH and 83 g L^−1^ WAS after 2 h operation. The copper electrode showed the highest catalytic activity for VFAs production.

The WAS oxidation was conducted in the electrochemical cell using different electrodes to investigate the effect of catalytic active surface species on the VFAs yield and volatile solids (VS) removal. The VFAs concentration, ammonia, and VS contents were measured after each experiment. The results of biomass conversion on different electrodes were compared at 35°C, 1.35 V for 2 h operation time in 0.2 M NaOH. The detailed information about the current density response is provided in Supplementary Figure S3. Copper electrodes showed the highest current density with the maximum of 30 mA cm^−2^, followed by nickel (14 mA cm^−2^) and stainless steel (4.5 mA cm^−2^). Figure 2 presents the VFAs concentration distribution (Figure 2A) and yield (Figure 2B) for the copper, stainless steel, and nickel electrodes. The highest VFAs concentration obtained was 454.2 ± 7.7 mg L^−1^ by utilizing copper electrodes which is 55 times higher than the VFAs concentration in the AD after 24 h digestion (100 mg L^−1^ per day, 8.3 mg L^−1^ in 2 h on average) (Fang et al., 2021). This result showed that the electrochemical conversion of WAS by using copper electrodes is more efficient for the VFAs production. Likewise, the highest VFAs yield of 48 mg g VS^−1^ was obtained for the copper substrate, followed by 21.4 ± 0.6 and 14.6 ± 0.5 mg gVS^−1^ for the nickel and stainless-steel electrodes, respectively. In contrast, a recent study utilizing AD under alkaline condition and addition of iron persulfate could only reached 382 mg gVS^−1^ after 4 days of fermentation time which translates to 8 mg gVS^−1^ in 2 h on average (He et al., 2019). It was observed that the VFAs distribution differs by using different electrodes. Acetic acid is the major component when SS is used as the working electrode. This can be explained by further oxidation of the fatty acid with a higher carbon number to acetic acid on the surface of the SS electrode. However, copper has shown C2-C5 components (e.g, acetic acid, propionic acid, butyric acid, isobutyric acid, valeric acid, and isovaleric acid) after 2 h of electrolysis. The ammonia concentration after electrochemical oxidation was evaluated for all three electrodes. The nickel electrode demonstrated the highest ammonia concentration compared to the other working electrodes. This observation agrees with the literature which shows that nickel is the active electrode for the electrochemically enhanced hydrolysis of urea to nitrogen in alkaline media (Lu and Botte, 2017, 2015). The summary of the electrode performance on the WAS oxidation is presented in Table 1.

**TABLE 1:** Comparison of the performance of the different electrodes on E-WAS at 1.35 V, 35°C and 2 h operation time.

Electrode	Copper	Nickel	Stainless steel
Total VFAs (mg L^−1^)	454.2±7.7	118.6±3.5	99.2±2.3
Yield VFAs (mg gVS^−1^)	48.7±1.4	21.4±0.6	14.6±0.5
Volatile solid removal (%)	21.8±0.7	17.7±0.5	13.2±0.4
Ammonia concentration (mg L^−1^)	126±3	250 ±4	168±2

### Cyclic voltammetry

**FIGURE 3:**
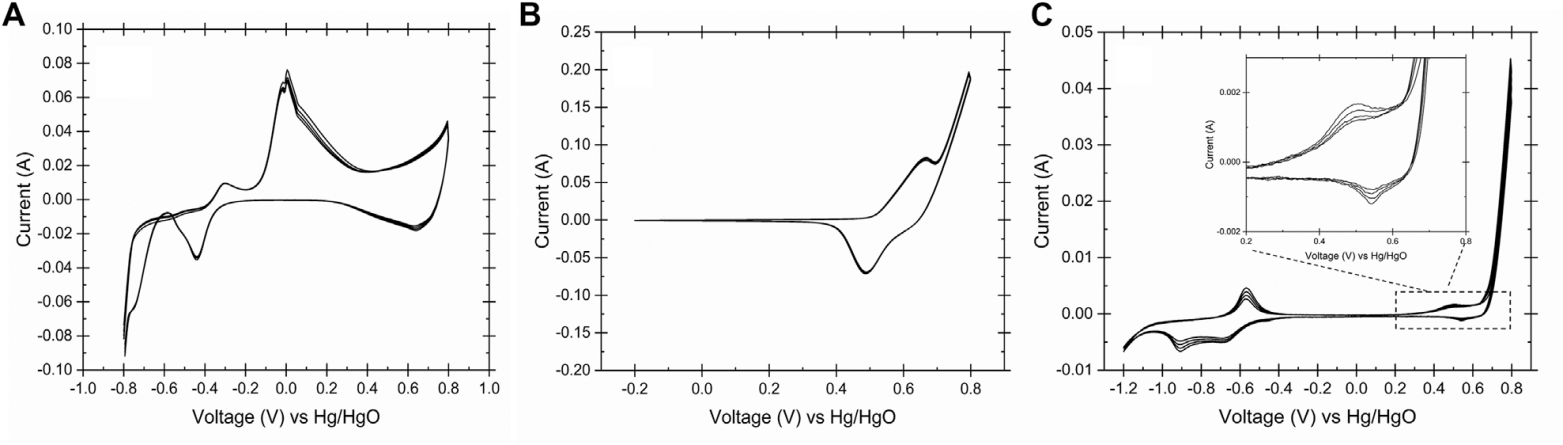
Cyclic voltammogram of the working electrodes obtained in 0.2 M NaOH, 25°C and scan rate 50 mV s^−1^, **(A)** copper, **(B)** Ni and **(C)** stainless steel 316. The CVs showed the identical oxidation/reduction peaks of copper, nickel and iron on the surface of working electrodes.

To identify the oxidation states on the surface of electrodes in alkaline media, the cyclic voltammetry technique was carried out for the SS, Ni, and copper electrodes in 0.2 M NaOH and 25°C. Figure 3 shows the CV curves of all the three electrodes used in this study. Figure 3A describes the oxidation states of the copper electrode in alkaline condition.

$$2Cu_{\left(s\right)}+2OH^{-}\to Cu2O_{\left(s\right)}+2e^{-}$$ (4)

$${\text{Cu}}_{2}{\text{O}}_{\left(\text{s}\right)}+2{\text{OH}}^{-}\to 2\text{Cu}{\text{O}}_{\left(\text{s}\right)}+{\text{H}}_{2}{\text{O}}_{\left(\text{l}\right)}+2{\text{e}}^{-}$$ (5)

$$\text{Cu}{\text{O}}_{\left(\text{s}\right)}+{\text{OH}}^{-}\to \text{CuOO}{\text{H}}_{\left(\text{s}\right)}+{\text{e}}^{-}$$ (6)

$$\text{Ni}{\left(\text{OH}\right)}_{2\left(\text{s}\right)}+{\text{OH}}^{-}\to \text{NiOO}{\text{H}}_{\left(\text{s}\right)}+{\text{H}}_{2}{\text{O}}_{\left(\text{l}\right)}+{\text{e}}^{-}$$ (7)

$${\text{Fe}}_{\left(\text{s}\right)}+2{\text{H}}_{2}{\text{O}}_{\left(\text{l}\right)}\to \text{Fe}{\left(\text{OH}\right)}_{2\left(\text{s}\right)}+2{\text{H}}^{+}+2{\text{e}}^{-}$$ (8)

$$\text{Fe}{\left(\text{OH}\right)}_{2\left(\text{s}\right)}+2{\text{OH}}^{-}\to {\text{Fe}}_{3}{\text{O}}_{4\left(\text{s}\right)}+4{\text{H}}_{2}{\text{O}}_{\left(\text{l}\right)}+2{\text{e}}^{-}$$ (9)

$$\text{Fe}{\left(\text{OH}\right)}_{2\left(\text{s}\right)}+2{\text{OH}}^{-}\to {\text{Fe}}_{3}{\text{O}}_{4\left(\text{s}\right)}+4{\text{H}}_{2}{\text{O}}_{\left(\text{l}\right)}+2{\text{e}}^{-}$$ (10)

$${\text{Fe}}_{3}{\text{O}}_{4\left(\text{s}\right)}+{\text{OH}}^{-}+{\text{H}}_{2}{\text{O}}_{\left(\text{l}\right)}\to 3\text{FeOO}{\text{H}}_{\left(\text{s}\right)}+{\text{e}}^{-}$$ (11)

Eqs 4–6 represents the copper oxidations reactions at -0.4 V, -0.1 V and 0.45 V vs Hg/HgO. It has been reported that the Cu (II)/Cu (III) redox couple is the catalytic active site for the glucose oxidation (Figiela et al., 2018). Due to the presence of the cellulose in the waste activated sludge and its conversion to glucose in aqueous media, it could be proposed that Cu (II) oxide film is a suitable catalyst for the WAS oxidation to volatile fatty acids. In basic solution, Ni (II) is oxidized to Ni (III) forming Ni oxyhydroxide according to Eq. (7). It is proposed that the NiOOH serves as a catalyst for the WAS oxidation at 0.5 V vs. Hg/HgO (Figure 3B). Eqs 8–11 describe the reactions on the surface of stainless-steel electrodes in 0.2 M NaOH. The transition of the Fe/Fe (II) and Fe (II)/Fe (III) on the surface of stainless steel at the redox potential range of -1 to -0.5 V vs. Hg/HgO (Figure 3C.) is noticed. Also, the Stainless-steel electrode showed similar redox couples in peak shape and position due to the formation of the Ni (II)/Ni (III) on the surface of stainless steel (Tiwari et al., 1991; Sultana et al., 2020). Although a durability study on the electrodes was not performed, the Pourbaix diagram of the Cu, Ni and SS confirmed the stability of the electrodes at the operating condition and their redox behaviors (Eqs 4–11, Davoodi et al., 2011; de Sousa et al., 2019). The proposed mechanism of the WAS oxidation on the surface of the working electrodes could be described as in reactions (12)–(15). At the cathode of the electrolysis cell, hydrogen evolves according to reaction (16). The gas chromatograph of the gas sample after E-WAS is shown in the Supplementary Figure S5.

$$\text{Ni}{\left(\text{OH}\right)}_{2\left(\text{s}\right)}+{\text{OH}}^{-}\to \text{NiOO}{\text{H}}_{\left(\text{s}\right)}+e^{-}$$ (12)

$$\text{NiOO}{\text{H}}_{\left(\text{s}\right)}+WAS\to \text{Ni}{\left(\text{OH}\right)}_{2\left(\text{s}\right)}+\text{VFAs}$$ (13)

$$\text{Cu}{\text{O}}_{\left(\text{s}\right)}+{\text{OH}}^{-}\to \text{CuOO}{\text{H}}_{\left(\text{s}\right)}+e^{-}$$ (14)

$$\text{CuOO}{\text{H}}_{\left(\text{s}\right)}+\text{WAS}\to \text{Cu}{\text{O}}_{\left(\text{s}\right)}+\text{VFAs}$$ (15)

$$2{\text{H}}_{2}{\text{O}}_{\left(\text{l}\right)}+2{\text{e}}^{-}\to {\text{OH}}^{-}+H_{2}$$ (16)

A similar reaction mechanism has been identified for organic compounds such as urea (Wang et al., 2012; Lu and Botte, 2017) glucose (Huang et al., 2014; Barragan et al., 2018) on the surface of the nickel-based and copper-based electrodes under alkaline conditions. Furthermore, this proposed mechanism was confirmed with the characterization on the surface of the copper electrode (*Cyclic voltammetry*).

### Copper oxide film characterization

**FIGURE 4:**
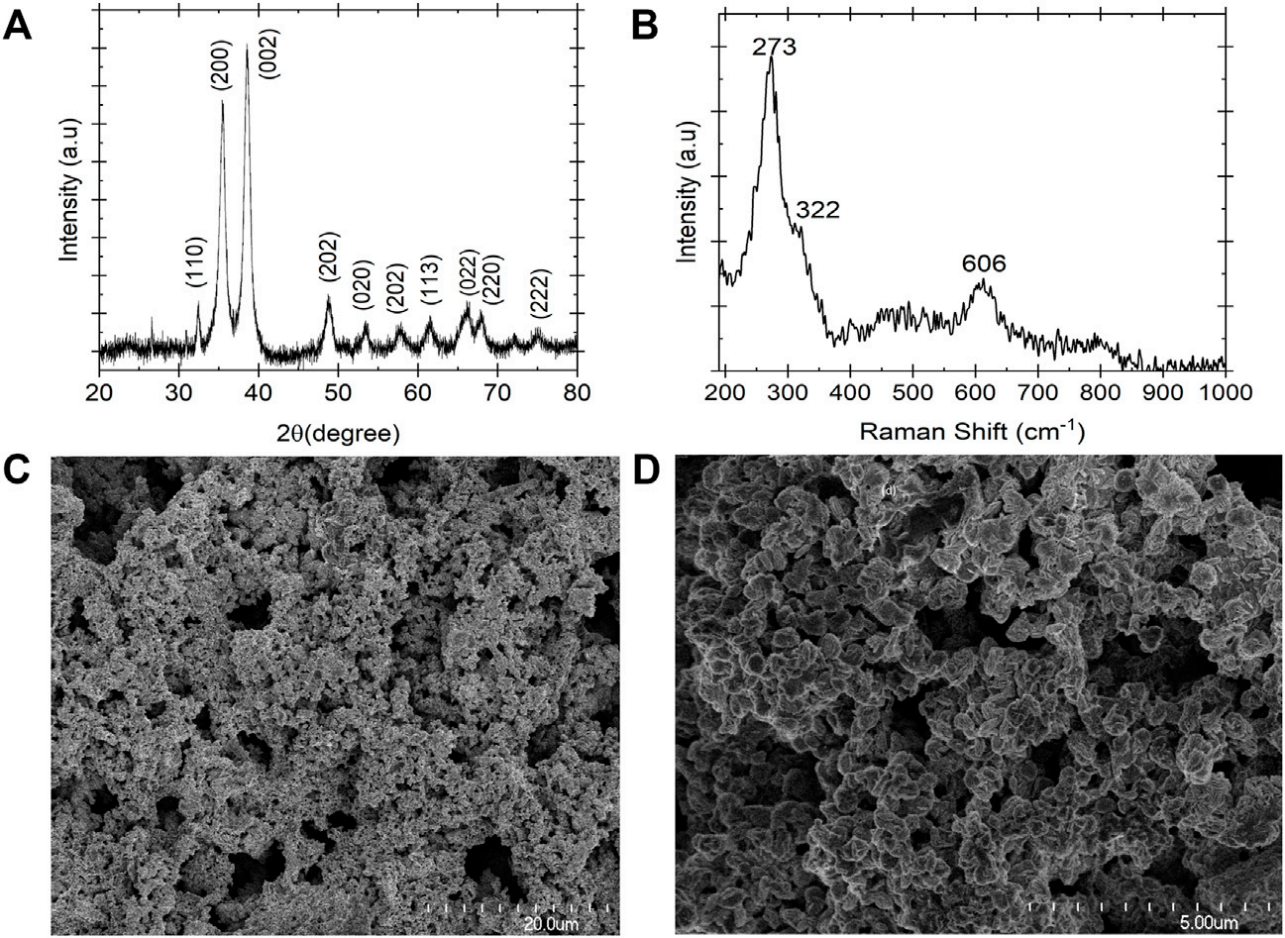
Characterization of copper oxide film formed on the surface of the copper substrate after electrochemical conversion of the solid samples in 0.2 M NaOH at 35°C. **(A)** XRD pattern **(B)** Raman Spectra and **(C,D)** FE-SEM image. The XRD and Raman spectra confirmed the copper oxide film formation, and the FE-SEM image shows the spherical arrangement of the film.

Figure 4A demonstrates the XRD pattern of copper (II) oxide film form on the surface of the copper film substrate after anodization at voltage 0.5 V vs. Hg/HgO. The average crystalline structure size of the CuO particles was estimated based on the Debye–Scherrer eq. D = K λ /β cos θ (17).Where the K is 0.94, λ is 0.15406 nm and β is the full width at the half maximum of the diffraction peak. Based on Eq 17 the calculated crystalline size of the CuO particles were evaluated to be 1.3–16.3 nm. According to the literature, the diffraction peaks positioned at 32.47, 35.56, 38.79, 46.22, 48.90, 53.46, 58.24, 61.55, 66.31, 68.13, 72.52, and 75.16 can be assigned to the monolithic CuO phase (Min Cha et al., 2017). All these peaks were indexed to the pure monoclinic CuO phase without impurities. To further confirm the CuO film formation on the surface of the copper foil Raman spectroscopy technique was carried out to investigate the chemical structure of the film (Figure 4B). The CuO film showed its characteristics Raman band at 273, 322, and 606 cm^−1^. The peak at 273 cm^−1^ corresponds to the Ag band and the peaks at 322 and 606 cm^−1^ correspond to the 2Bg band. The frequencies of the Raman bands observed in this study are in agreement with the literature (Deng et al., 2016; Min Cha et al., 2017; Su et al., 2019). Raman of the copper electrode after electrolysis confirmed the formation of the CuO layer on the surface of the copper substrate and this is consistent with the XRD and CV results. To observe the morphology and microstructure of the electrode, SEM was performed on the copper oxide film. Figures 4C,D show a tight pack of spherical shape particles arrangement formed on the surface of the working electrode.

### Effect of applied potential and temperature on VFAs production

**FIGURE 5:**
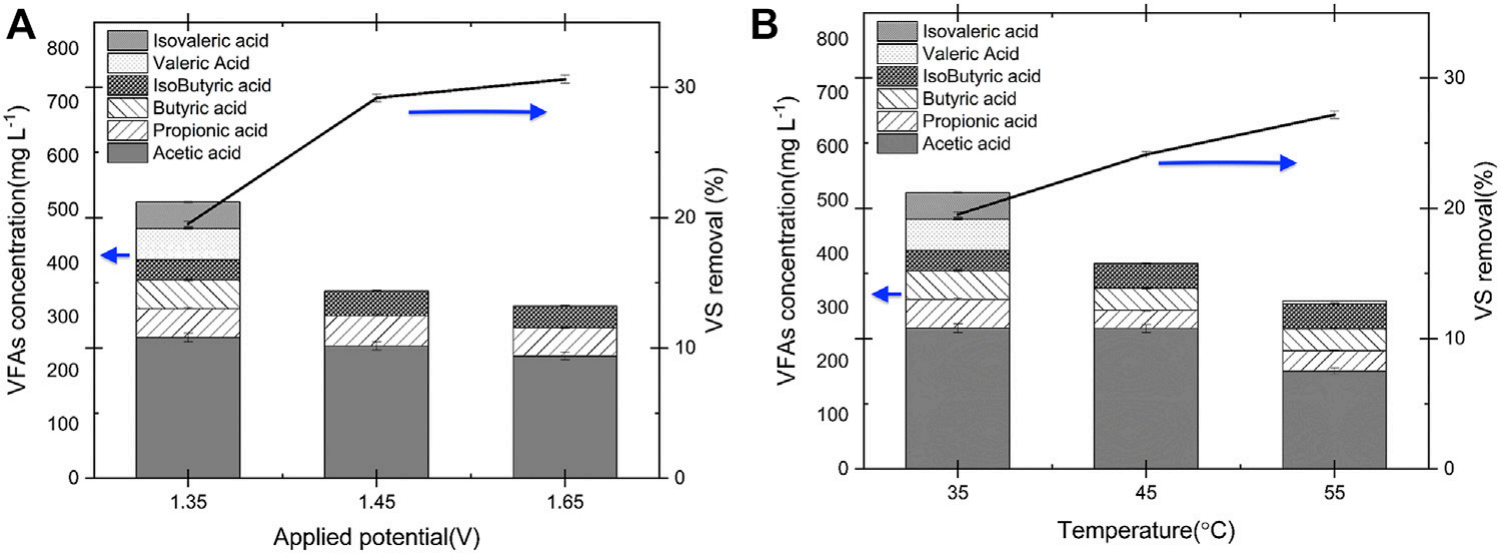
Comparison of VFAs concentrations during E-WAS with Cu electrode at 35°C in 0.2 M NaOH and 83 g L^−1^ WAS after 1 h operation **(A)** different applied cell potentials. **(B)** at 1.35 V and 35–55°C operation temperature. VS. removal increased at higher operating temperature and applied potential. However, VFAs concentration significantly decreased. Highest total VFAs concentration was obtained at 35°C and 1.35 V.

The effect of applied potential and temperature on VFA production was investigated using the copper electrodes (as they show highest yield to VFAs production when compared to nickel and stainless steel) and the results are shown in Figures 5A,B. The VS. removal efficiency increased from 19 to 30% by increasing the cell potential from 1.35 to 1.65 V, on the other hand the total VFAs concentration decreased by 37.6%, from 515.85 mg L^−1^–321.0 mg L^−1^ (see Figure 5A). This shows that operating at higher applied potentials increases the biomass oxidation rate, including the oxidation of VFAs. Likewise, a similar trend was identified for the VFAs concentration with operating E-WAS at higher operating temperatures (Figure 5B). The VFAs concentration decreased substantially from 514.8 to 340 mg L^-l^ by increasing the operating temperature from 35 to 55°C. Moreover, working at a lower temperature is more economical due to lower energy consumption. Therefore, to increase VFAs accumulation in the electrolyte it is better to operate at low applied potentials (1.35 V) and temperatures (35°C). Also, as reported in *Effect of applied potential and temperature on VFAs production* the VFAs concentration after E-WAS using of copper electrodes reached 454.2 mg L^−1^ (Table 1), that is, longer E-WAS operation time leads to decrease in VFAs accumulation due to oxidation on the fatty acids. Therefore, an optimization process that includes production/separation after 1 h of operation in E-WAS is proposed to enhance the VFAs production in a continuous process. The process should be optimized with continuous separation of products to maximize yields. On the other hand, the highest VS. removal of 30% was obtained at 1.65 V applied cell potential (35°C, Figure 5A) after 1 h electrochemical oxidation with 80% less sodium hydroxide concentration (0.2 M NaOH) which is more efficient than conventional alkaline treatment of WAS after 24 h operation (55%, 1 M NaOH) (Jafari and Botte, 2021).

### Effect of electrolysis on the WAS morphology

**FIGURE 6:**
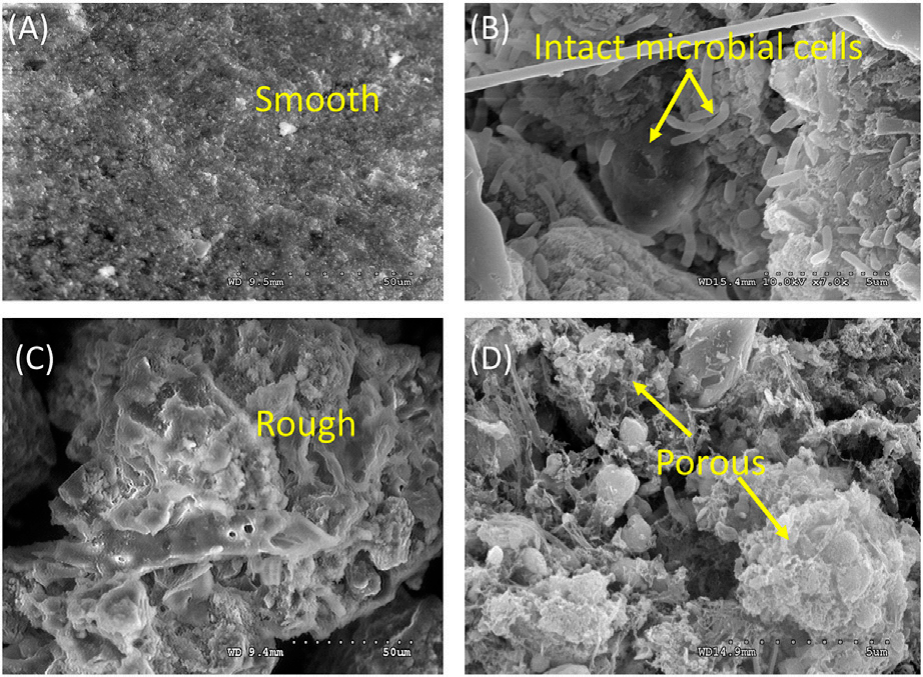
**(A,B)** Rw WAS morphology and **(C,D)** after 2 h E-WAS operation at 1.35 V 35°C in 0.2 M NaOH and 83 g L^−1^ WAS. The morphology changed from the smooth surface with the presence of intact microbial cells to the rough, porous structure which represents damage in the microbial cell and sludge flocs.

Floc disruption and cell lysis of sludge took place during the E-WAS process as demonstrated by the change in the morphology and microstructure of sludge. Scanning electron microscope was used to observe the raw sludge and the sludge treated by E-WAS. Field-emission Scanning electron microscope (SEM) images show the morphology of sludge particles before and after E-WAS, the image of the raw sludge sample (Figures 6A, B show mainly irregular and smooth surfaces with the presence of intact microbial cells (yellow arrow). However, in reacted sludge (Figures 6C, D), the collapse of sludge flocs formed a more rough, porous, and shrivelled structure (marked by arrows). Furthermore, the microbial cells are not presented due to sludge disintegration.

## Energy consumption and perspectives

Table 2 presents the energy consumption of the E-WAS (per kg of dried TS and kg of VFA) for the SS, Ni and Cu electrodes calculated based on the results provided in Table 1. It has been reported that the energy consumption for WAS treatment should be less than 0.2 KWh kg TS^−1^ for achieving self-efficiency for a conventional WWTP (Cano et al., 2015). The energy consumption for our electrochemical treatment at the lab scale system was 0.001, 0.005 and 0.37 kWh kg^−1^ TS for SS, Ni, and Cu respectively at 35°C, 1.35 V and 2 h of electrolysis which are significantly lower than the target (0.2 kWh kg^−1^ TS). The energy consumption per kg of VFAs produced is also within the lowest values reported in the literature (Zeng, et al., 2022) while there is still room for optimization. It is envisioned that the E-WAS can be combined with electrodialysis to achieve separation and recovery of multiple value products including VFA, and ammonia, simultaneously (Zhang and Angelidaki, 2015). The process could also be integrated with struvite precipitation by adding magnesium salt to the solution to recover phosphorous which could also be present in the solution (Lorick et al., 2020; Li et al., 2021; Saabas and Lee, 2022). Future studies should consider electrode optimization, multiple separation of products, optimization of polarity switching time, voltage, electrode durability and scale up to access full techno-economic analysis of the process.

**TABLE 2:** Comparison of the energy consumption of E-WAS for VFA production implementing SS, Ni and Cu electrodes at 1.35V, 35°**C** and 2 h operation time.

Electrode	Energy consumption per kg of dried solids (kWh kg TS^−1^)	Energy consumption per kg of VFA (kWh kg VFA^−1^)
SS	0.001 ± 0.002	0.091 ± 0.002
Ni	0.005 ± 0.002	0.429 ± 0.025
Cu	0.037 ± 0.002	1.354 ± 0.267

## Conclusion

In this study, the electrolysis of waste active sludge (E-WAS) to VFAs production with transition metal electrodes in alkaline media was demonstrated and investigated. The results demonstrate that applying 1.35 V with a polarity switch of 10 s for short operating times (2 h) using copper electrodes in comparison to the nickel and stainless steel not only could significantly reduce the total volatile solid, but also it could successfully produce higher VFAs concentrations. The findings in this study demonstrated that copper oxide is an active catalyst for valorization of WAS by producing VFAs with higher production rate in comparison to the traditional anaerobic digestion. Additionally, E-WAS could simultaneously destroy the microbial cells in the WAS. On the other hand, nickel electrode showed a good catalytic activity for ammonia production through E-WAS. The total energy consumption of 0.037 ± 0.002 kWh kg TS^−1^ E-WAS was obtained for Cu electrode which is much lower than the target cited in the literature for achieving self-efficiency for a conventional WWTP (0.2 kWh kg TS^−1^). To further investigate the reaction mechanism a comprehensive study on the WAS model compounds needs to be performed. The VFAs yield could be further improved by utilizing a bimetallic electrode material. VFAs and ammonia recovery from the electrolyte by integrating electrodialysis process is recommended for future works. Further investigation on electrode optimization and stability tests should be performed in future studies. This work provides new venues for electrochemical digestors which could enable process intensification in municipal wastewater treatment plants.

## Data Availability

The original contributions presented in the study are included in the article/Supplementary Material further inquiries can be directed to the corresponding author.
