# Supplementary material for: Electrochemical valorization of waste activated sludge for short-chain fatty acids production
Source: Front Chem. 2022 Aug 30;10:974223. doi: 10.3389/fchem.2022.974223 (PMC9469876; doi:10.3389/fchem.2022.974223)
Supplement: Supplementary file 1 [file DataSheet1.DOCX]

Supplementary Material

**Electrochemical Valorization of Waste Activated Sludge for Short-Chain Fatty Acids Production**

Maasoomeh Jafari^1^, Gerardine G. Botte^1*^

^1^ Chemical and Electrochemical Technology and Innovation Laboratory, Department of Chemical Engineering, Edward E. Whitacre Jr. College of Engineering, Texas Tech University, Lubbock, TX, USA

*** Correspondence:**Gerardine G. Botte
[gerri.botte@ttu.edu](mailto:gerri.botte@ttu.edu)

# Supplementary Figures and Tables

## Supplementary Figures

**
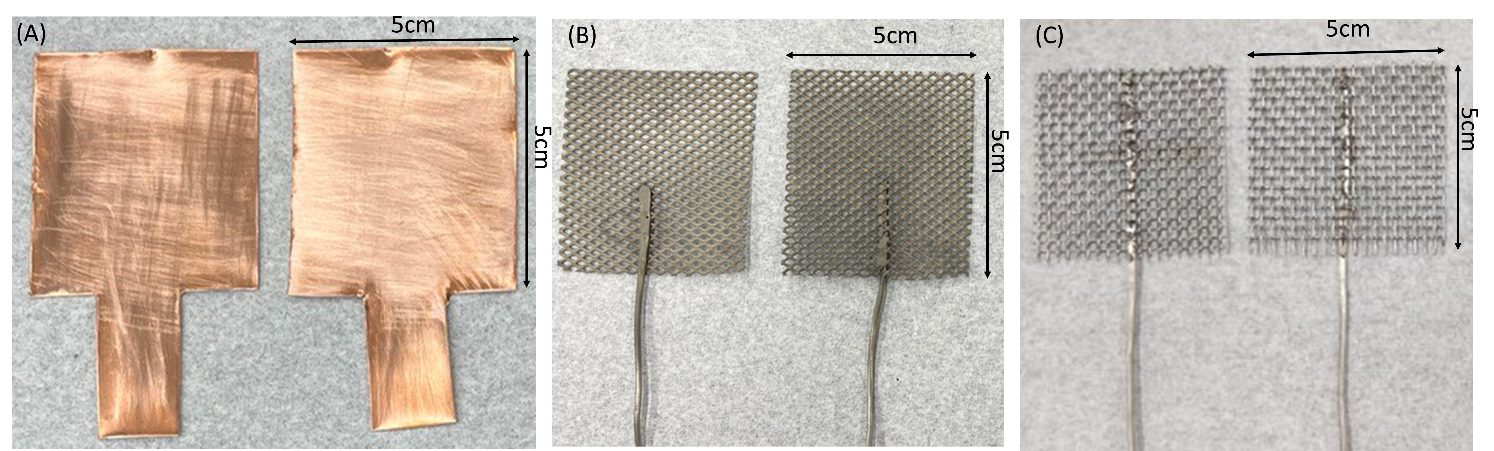
Electrodes dimensions, electrochemical cell, and current density profiles**

**Supplementary Figure 1**. Electrodes’ dimension **(A)** copper electrode **(B)** nickel electrode **(C)** Stainless steel mesh


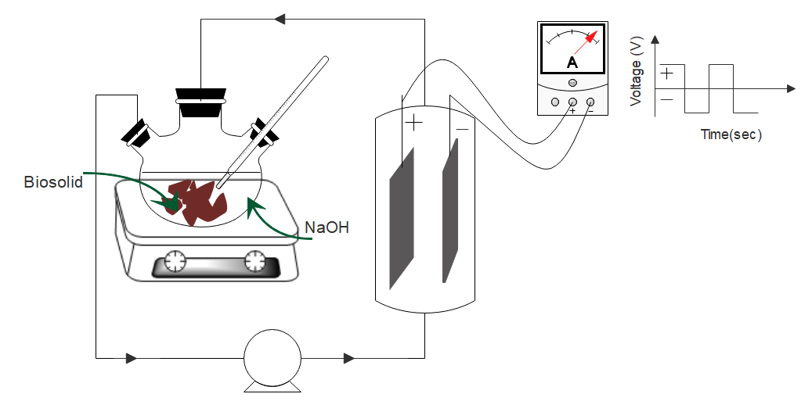


**Supplementary Figure 2**. Schematic of the sludge electrolysis system for 600 ml feed volume (83 g L^-1^ solid concentration and 0.2 M NaOH), flowrate 401 ml min^-1^, temperature range of 35-55 ℃ with switch polarity technique (three different voltages were investigated: 1.35 V, 1.45 V, and 4.65V, 10 s pulse time). The feed mixture was mixed for 5 min before feeding to the electrolysis cell to assure homogeneous input (Jafari and Botte, 2021)

**
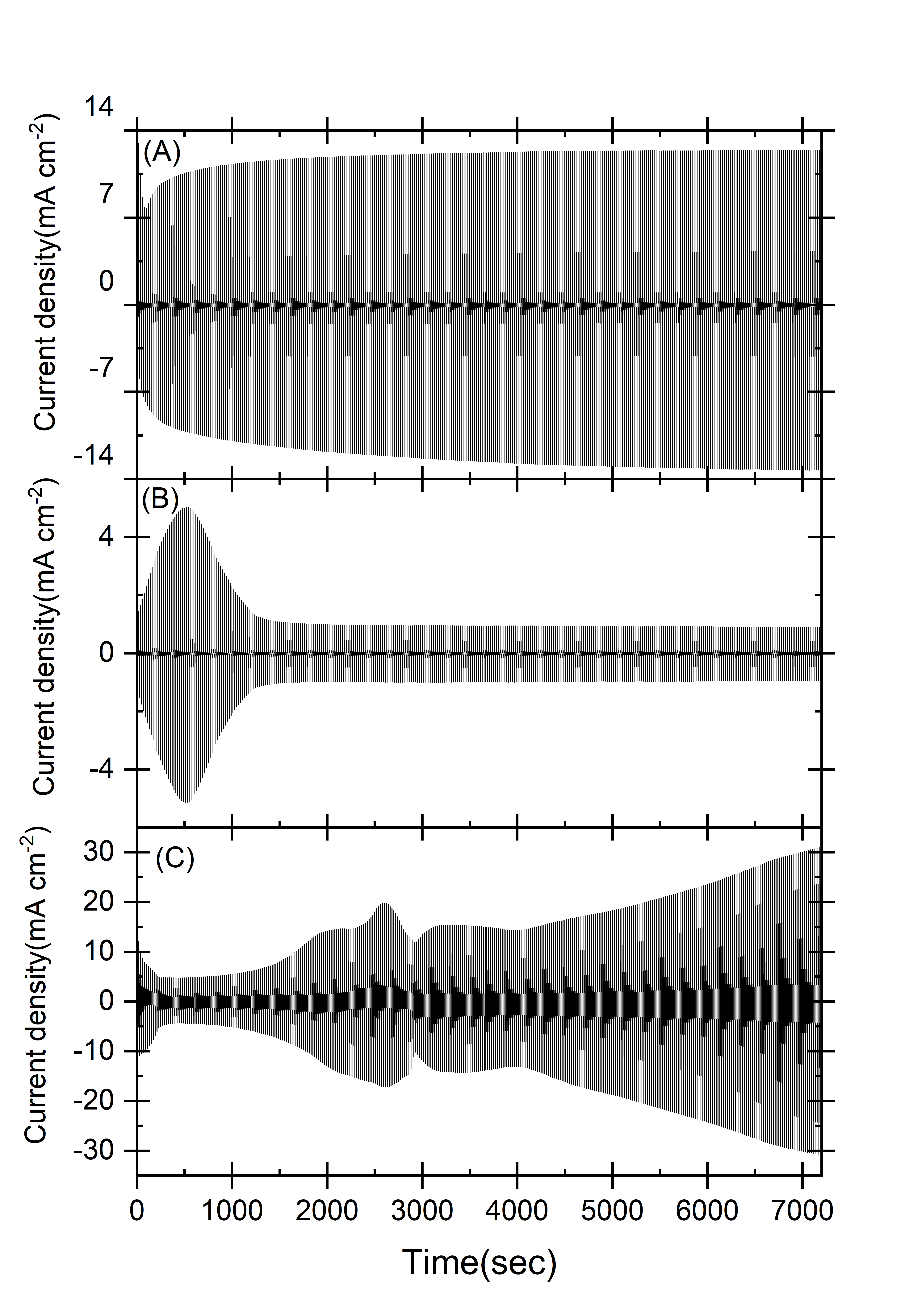
**

**Supplementary Figure 3.** Current profiles of electrochemical cell at 35 ºC and 1.35 V for **(A)** nickel electrodes **(B)** stainless electrodes **(C)** copper foils. The highest current density was obtained for copper electrodes followed by nickel and stainless steel

**2. Critical point drying procedure**

The solid samples were put into the sample holder (stainless-steel 316, B5222 standard specimen holder). The sample holder lid was closed and immersed in the 20 mM phosphate buffer solution and 5% glutaraldehyde solution overnight ($\sim$ 12h) (Supplementary Figure 4). The samples were rinsed with the phosphate buffer solution 20 mM three times for 10 mins each. Then the samples were rinsed with different ethanol concentrations (25%, 50%, 75%, 85% 95% and 100%). The detailed information about the rinsing time with alcohol is summarized in Supplementary Table. After rinsing the samples with ethanol, the sample holder was put into the critical drying instrument. First, the samples were purged with liquid carbon dioxide (LCO_2_, Airgas) to remove all the alcohol residue in the samples then the sample were dried under LCO_2_ at 1073 psi and 31.1 ºC for 15 mins.


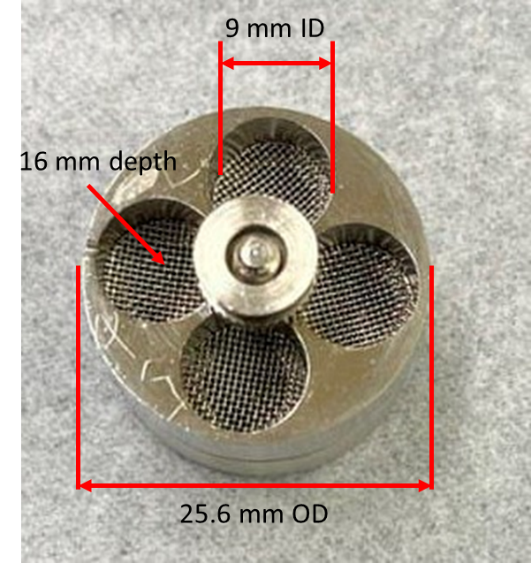


**Supplementary Figure 4**. Critical point drying sample holder schematic


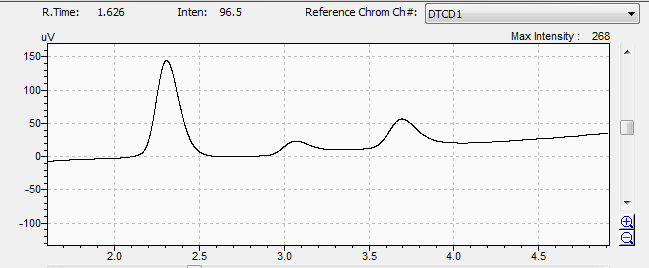


**O_2_**

**H_2_**

**N_2_**

**Supplementary Figure 5.** Gas chromatograms of the gas samples after E-WAS at 1.35 V and 35 ºC

Supplementary Table 1. Biomass conditioning procedure for the critical point drying

| Rinsing Solution | Time (mins) |
| --- | --- |
| 25% ethanol | 10 |
| 50% ethanol | 10 |
| 75% ethanol | 10 |
| 85% ethanol | 10 |
| 95% ethanol | 10 |
| 95% ethanol | 10 |
| 100% ethanol | 15 |
| 100% ethanol | 15 |
| 100% ethanol | 15 |
| 100% ethanol | 15 |

**3. References**

Jafari, M. and Botte, G.G. (2021) ‘Electrochemical treatment of sewage sludge and pathogen inactivation’, *Journal of Applied Electrochemistry*, 51(1), pp. 119–130. doi:10.1007/s10800-020-01481-6.

.
